# Three-year epidemiological analysis of penetrating ocular traumatic endophthalmitis

**DOI:** 10.1097/MD.0000000000038308

**Published:** 2024-07-05

**Authors:** Xiaotong Zhuang, Bo Fu, Jie Dong, Qiushi Liu, Shiqiao Jia, Li Xu

**Affiliations:** a Department of Ophthalmology, The Fourth People’s Hospital of Shenyang, China Medical University, Shenyang, China.

**Keywords:** endophthalmitis, intraocular foreign body, penetrating eye injuries, visual acuity

## Abstract

The characteristics of patients with endophthalmitis due to penetrating ocular trauma are still limited. The aim of the study was to fill these gaps among Chinese population. This retrospective study included patients diagnosed as penetrating ocular traumatic endophthalmitis between January 2016 to December 2018. During the past 3-year period, a total of 201 patients with antecedent penetrating eye injuries were evaluated. Of which, 42 (20.9%) patients presented a clinical course compatible with acute infectious endophthalmitis. 39 (92.86%) patients were males, and 15 (35.71%) patients had mechanical injuries from intraocular foreign body (IOFB), the rate of endophthalmitis due to IOFB was 13.43%, higher to the rate among patients without IOFB (7.46%). The duration between injury occurrence and endophthalmitis onset was 1 day in 10 (23,80%) patients; 2 to 7 days in 31 (73.80%) patients, and 7 to 14 days in 1 (2.38%) patient. After 1 year follow-up, best corrected visual acuity (BCVA) better than 20/400 was observed in 15 (35.71%) patients, counting fingers and hand move in 17 (40.48%) patients, light perception in 5 (11.9%) patients and no light perception in 5 (11.9%) patients, respectively. Patients with promising outcomes had better initial BCVA at baseline (*P* < .001). Endophthalmitis is a severe ocular infectious condition that may lead to irreversible vision loss. A greater attention must be paid to penetrating eye injuries within males, who had poor BCVA at baseline, particularly with obvious IOFB.

## 1. Introduction

Penetrating eye injury is one of the most devastating attacks to visual acuity (VA), especially complicated with endophthalmitis and intraocular foreign body (IOFB).^[1,2]^ Endophthalmitis is a severe infectious ocular disease involving vitreous or aqueous fluids and other intraocular tissues that can result in blindness, and requires immediate injection of intravitreal antibiotics or vitrectomy.^[3,4]^ The incidence of post-traumatic endophthalmitis will be approximately 16.5%.^[5,6]^ The pathogen is typically related to the periocular cutaneous microbiota. There were also some atypical agents reported including Bacillus cereus.^[4,7]^ To date, endophthalmitis rates and risk factors following penetrating eye injuries in the Medicare population has not been reported. Through retrospective analysis of patients with penetrating eye injuries in our hospital in the past 3 years, we reveal the risk factor of endophthalmitis and best corrected visual acuity (BCVA) prognosis among patients with penetrating eye injuries.

## 2. Methods

This retrospective study reviewed the characteristics of patients with diagnosis of penetrating eye injuries who were treated in the emergency department from January 2016 to December 2018 at Shenyang fourth people`s hospital. The demographic information including age, gender, and medical histories such as traumatic agents, time from trauma to endophthalmitis onset, culture results, treatment, initial BCVA and prognosis were collected.

For patients with penetrating eye injuries, our study followed a principle of immediate admission for emergency surgery. Before surgery, all patients underwent orbital 3D computed tomography to assess the presence of IOFB. Primary closure procedure was performed, followed by vitrectomy and silicone oil filling if necessary, and removed the IOFB during the first surgery. Intravitreal antibiotics were used at the end of the surgery.^[7]^ The standard intravitreal injection is 0.05 mL Vancomycin (1 mg) combined with 0.05 mL Ceftazidime (2.25 mg). The information on ophthalmic checks included BCVA, chemosis, hyperemia, ocular pain, eyelid edema and intraocular inflammatory signs, such as anterior chamber reaction, hypopyon, fibrin, and vitreous opacity.^[3, 7, 8]^ The existence of IOFB was evaluated carefully. This study was conducted according to the Declaration of Helsinki. The ethics committee of Shenyang fourth people hospital approved this study (0030/2019).

### 2.1. Statistical analysis

All statistical analyses were performed using SPSS 23.0 software (IBM SPSS Statistics for Windows, Version 23.0, Armonk). The Shapiro–Wilk test of Normality was used to determine whether the baseline values of the dependent variables had a normal distribution. According to normality testing, categorical values were described as frequencies and percentages, whereas continuous variables were reported using appropriate central tendency and dispersion measures. Chi-square test or Mann–Whitney *U* test rank and contrast analysis was used for comparison between groups according to outcomes. A value of *P* < .05 was accepted as statistically significant.

## 3. Results

Totally, 201 patients underwent penetrating eye injuries during the three years with or without an IOFB were included. Overall, 42 (20.9%) patients presented a clinical course compatible with acute infectious endophthalmitis. The mean age of the patients was 49.67 ± 10.38 years (range 23–73 years). Of which, 39 (92.86%) patients were males, and 15 (35.71%) patients presented with IOFB. The traumatic agents responsible for penetrating eye injuries were metal in 7 (46.67%) patients, stone in 5 (33.33%) patients, wood in 1 (6.67%) patient, plastic in 1 (6.67%) patient, and caterpillar in 1 (6.67%) patient. The rate of endophthalmitis with IOFB among penetrating eye injuries patients was 13.43%, while the rate without IOFB was 7.46% (Table 1). There were 7 patients with traumatic cataract at baseline. Moreover, in terms of wound zone, we divided the anterior ocular into 3 zones in this study specifically. There were 98 patients with the center of 3 mm in diameter of the cornea (zone I), 65 patients with the posterior 1.5 mm next to zone I (zone II), and 38 patients with the border of zone II to 6 mm posterior of the corneoscleral limbus.

**Table 1:** Epidemiological and trauma parameters of the 42 endophthalmitis patients due to penetrating eye injuries.

Variable	No of patients (%)
Sex	
Male	39 (92.86)
Female	3 (7.14)
Total	42 (100)
Composition of intraocular foreign body	
Metal	7 (46.67)
Stone	5 (33.33)
Plastic	1 (6.67)
Wood	1 (6.67)
Caterpillar	1 (6.67)
Total	15 (100)
Time from trauma to endophthalmitis onset	
Less than 1 day	10 (23.80)
Between 2 and 7 days	31 (73.80)
Between 7 and 14 days	1 (2.38)
Traumatic cataract	7 (100)
Results of positive cultures	
Staphylococci	7 (43.75)
Streptococcus sanguis	2 (12.5)
Enterococcus aureus	1 (6.25)
Acinetobacter rubens	1 (6.25)
Pseudomonas aeruginosa	1 (6.25)
Bacillus cereus	3 (18.7)
Corynebacterium	1 (6.25)
Total	16 (100)
BCVA (baseline/1 year later)	
NLP	4 (9.52)/5 (11.90)
LP	18 (42.86)/5 (52.38)
CF-HM	12 (28.57)/17 (40.48)
20/1000-20/20	8 (19.05)/15 (35.71)

BCVA = best corrected visual acuity, CF = counting fingers, HM = hand motion, IOFB = intraocular foreign body, LP = light perception, NLP = no light perception.

The duration between trauma and endophthalmitis onset was one day in 10 (23.80%) patients, 2 to 7 days in 31 (73.80%) patients, 7 to 14 days in 1 (2.38%) patient, and there was no patient over 15 days (Table 1).

Among the 42 patients diagnosed as acute infectious endophthalmitis, 16 (38.09%) patients presented positive cultures outcomes. Three of these cultures were positive only in aqueous culture (18.75%), 11 only in vitreous culture (68.75%), and 2 in both aqueous and vitreous culture (12.5%). The responsible etiological agents were coagulase-negative staphylococci in 7 cultures (43.75%) and streptococcus sanguis in 2 cultures (12.5%). Other cultures were positive for enterococcus aureus (1 patient, 6.25%), acinetobacter rubens (1 patient, 6.25%), pseudomonas aeruginosa (1 patient, 6.25%), bacillus cereus (3 patients, 18.75%), and corynebacterium (1 patient, 6.25%, Table 1).

Among the 42 patients with acute infectious endophthalmitis, 41 patients received cornea wound suture and 1 patient had a self-sealed wound. Furthermore, 26 (61.9%) patients (26 eyes) received vitrectomy and 21 eyes were filled with silicon oil during the surgery. The others received anterior chamber irrigation and intravitreal antibiotics injection without vitrectomy.

Initial BCVA during endophthalmitis was between 20/20 and 20/1000 in 8 (19.05%) patients, counting fingers, and hand move in 12 patients (28.57%), light perception (LP) in 18 (42.86%) patients and no light perception (NLP) in 4 (9.52%) patients. Four patients experienced eye enucleation. One year after endophthalmitis, BCVA better than 20/1000 were found in 15 (35.71%) patients, counting fingers and hand move in 17 (40.48%) patients, LP in 5 (11.9%) patients, and NLP in 5 (11.9%) patients, and 4 patients underwent enucleation with NLP (Table 1). Only initial VA was associated with prognostic outcome (*P* < .001, Table 2).

**Table 2:** The relationship between final best corrected visual acuity and indicators.

	20/1000-20/20	CF-HM	LP	NLP	χ^2^ value	*P* value
With IOFB	4	6	2	3	0.706	.951
Without IOFB	11	11	3	2		
Time from trauma to endophthalmitis onset						
1 day	3	4	1	2	5.038	.754
2–7 days	12	12	4	3		
8–14 days	0	1	0	0		
Cultures outcomes						
Positive	6	5	2	3	3.232	.520
Negative	9	12	3	2		
Initial VA						
20/1000-20/20	8	0	0	0	51.336	<.001
CF-HM	5	7	0	0		
LP	2	10	5	1		
NLP	0	0	0	4		
With traumatic cataract	4	1	1	1	0.612	.743
Without traumatic cataract	13	12	8	2		

CF = counting fingers, HM = hand motion, IOFB = intraocular foreign body, LP = light perception, NLP = no light perception, VA = visual acuity.

## 4. Discussion

Endophthalmitis is still a severe inflammatory ocular disease with profound vision loss that may become irreversible and requires immediate treatment.^[3,4]^ An epidemiological analysis plays key role in better understand the associations among the traumatic agents, time from trauma to endophthalmitis onset, culture results and VA and to establish a visual prognosis, treatment method and appropriately treat similar cases. This study comprehensively evaluated characteristics of endophthalmitis after penetrating eye injuries in China.

In current study, the prevalence of post-penetrating trauma endophthalmitis was 20.9% during the past 3 years, which was higher than the variable prevalence rates of 1% to 17% having been reported in the literature.^[8,9]^ This may be due to the environment when the patients being injured. In this study, we provided prophylactic intravitreal antibiotics at the end of the first surgery. The high prevalence of endophthalmitis also revealed that prophylactical use of vancomycin did not reduce the incidence of endophthalmitis effectively after penetrating eye injuries. Meanwhile, postoperative hemorrhagic occlusive retinal vacuities associated with prophylactical use of intracameral vancomycin were reported during cataract surgery.^[10–12]^ More advanced managements are needed urgently to prevent endophthalmitis after penetrating eye injuries. Furthermore, only three patients with acute endophthalmitis were females in our study, corroborating previous reports of higher prevalence in males.^[13]^ In that case, endophthalmitis symptoms started acutely, within 14 days after the ocular trauma and within 7 days in majority of the patients (73.8%). Infectious endophthalmitis after penetrating eye injuries has higher virulence and severity, which should be paid attention to within 2 weeks after trauma.

Of note, not all patients with symptoms of endophthalmitis had IOFB, only 15 (35.71%) patients had IOFB in our study. Furthermore, the rate of endophthalmitis with IOFB was 13.43%, higher than the rate (7.46%) among patients without IOFB, and IOFB did not increase the percentage of endophthalmitis in penetrating eye injuries patients. These maybe related to the attention was commonly paid by doctors to the patients with IOFB, and resulting early treatments of those patients. However, the severity of patients with IOFB was commonly missed by doctor compared with those with IFOB. When considering patients with IOFB, the rate of NLP 1 year after endophthalmitis was even higher (20%). The incidence of post-traumatic endophthalmitis may not be related to IOFB, but to traumatic characteristics and the agents related to ocular trauma.

Nearly 61.9% of 42 patients presented negative culture outcomes, which was incompatible with the results of the endophthalmitis vitrectomy study (EVS), which is a distinct disease compared to trauma-related endophthalmitis.^[7]^ The low rate of positive culture test results maybe contributed to the antibiotics used at the first surgery and the culture method. Four patients whose culture results were Bacillus Cereus (2 patients) and Staphylococcus Epidermidis (2 patients) exhibited VA of NLP with cornea dissolved and received eye enucleation. The sensitivity of vitreous culture was higher than aqueous culture (30.95% vs 11.90%), the same with the study by Nakayama,^[14]^ in contrast to the survey by Baza,^[15]^ probably due to differences in the characteristics of trauma-related endophthalmitis and the material collected. In current study, positive culture outcomes indicated only one pathogen in 38.09% of our patients, same with acute endophthalmitis after cataract surgery^[3]^ and post-intravitreal anti-vascular endothelial growth factor (anti-VEGF) injection,^[2]^ which are also characterized by a single pathogen. However, penetrating eye injuries may be associated with much more virulent and severe infections and the trauma agent is also the reason for the irreversible vision loss.

Through risk factor analysis, we know that the prognosis of endophthalmitis after penetrating trauma is only related to initial VA. Only 20% had VA better than 20/1000, further indicating severe infection with a poor prognosis. We report the poor prognosis of visual acuity in endophthalmitis after penetrating trauma, with an evaluation of the causative agents and microbiological analysis. Our findings confirmed that even with aggressive treatment, acute infectious endophthalmitis had a severe prognosis and the prognosis was even poorer in penetrating eye injuries.

This study has some limitations. It is a retrospective study and the inclusion of a single hospital’s experience. The lack of access to prompt ophthalmological emergency care in the areas with low medical coverage contributes to the time lapse between trauma and evaluation. Further studies are needed to evaluate antibiotic efficacy and improve antibiotic treatment to reduce infectious endophthalmitis after penetrating eye injuries.

## 5. Conclusion

Endophthalmitis is a severe intro-ocular infectious condition that may lead to irreversible vision loss. More attention should be paid to penetrating eye injuries endophthalmitis, particularly in males, patients with bad VA at baseline, and without IOFB.

## Author contributions

**Conceptualization:** Xiaotong Zhuang.

**Data curation:** Xiaotong Zhuang, Bo Fu, Jie Dong, Qiushi Liu, Shiqiao Jia, Li Xu.

**Formal analysis:** Bo Fu, Shiqiao Jia.

**Funding acquisition:** Bo Fu.

**Methodology:** Jie Dong.

**Project administration:** Li Xu.

**Resources:** Shiqiao Jia.

**Validation:** Jie Dong, Qiushi Liu.

**Visualization:** Qiushi Liu.

**Writing – original draft:** Xiaotong Zhuang.

**Writing – review & editing:** Li Xu.
